# Supplementary material for: Gate-controlled conductance switching in DNA
Source: Nat Commun. 2017 Feb 20;8:14471. doi: 10.1038/ncomms14471 (PMC5321735; doi:10.1038/ncomms14471)
Supplement: Supplementary Information — Supplementary Figures, Supplementary Tables, Supplementary Notes and Supplementary References [file ncomms14471-s1.pdf]

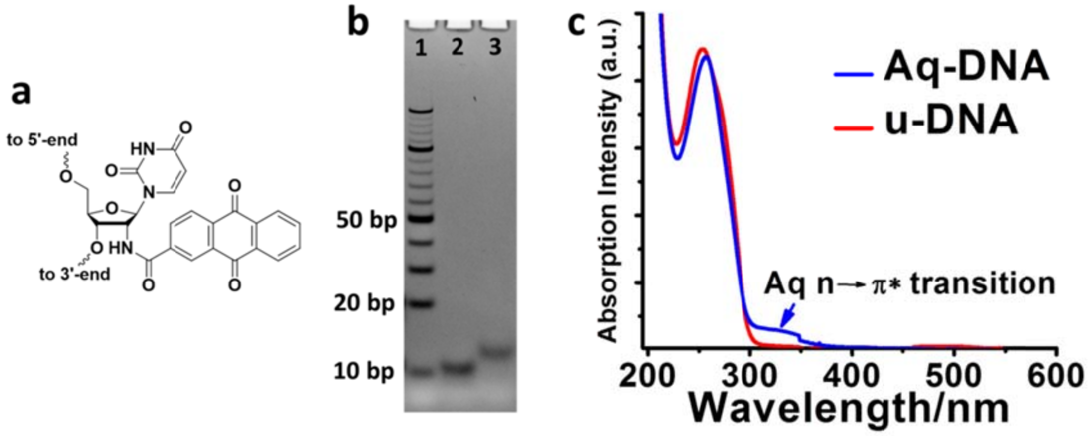

Supplementary Figure 1 | Structure, Nondenaturing PAGE gel electrophoresis and UV-Vis absorption spectra of Aq-DNA and u-DNA. a, Anthraquinone (Aq) moiety attached to the DNA backbone. The Aq moiety stacks with adjacent bases while the uracil moiety is located in the minor groove[1], allowing charge transport through the overlapping π–orbitals. **b,** The formation of double-helical DNA was confirmed by nondenaturing polyacrylamide gel electrophoresis. Column 2 is u-DNA. Column 3 is Aq-DNA. Longer DNA sequences have slower mobility as indicated by column 1 for the DNA ladder sample (DNA mixture consisting of 10 bp-200 bp dsDNA in 10bp intervals, also referred to as DNA ruler). Aq-DNA has a slight slower mobility than u-DNA, due to the extra Aq moiety. **c,** Both spectra have strong absorption at 260 nm, which is a typical absorption peak for DNA. The Aq-DNA has a weak absorption at around 340 nm, due to the anthraquinone n to π* transition absorption. The UV-Vis spectra confirmed the anthraquinone was successfully introduced into the DNA.

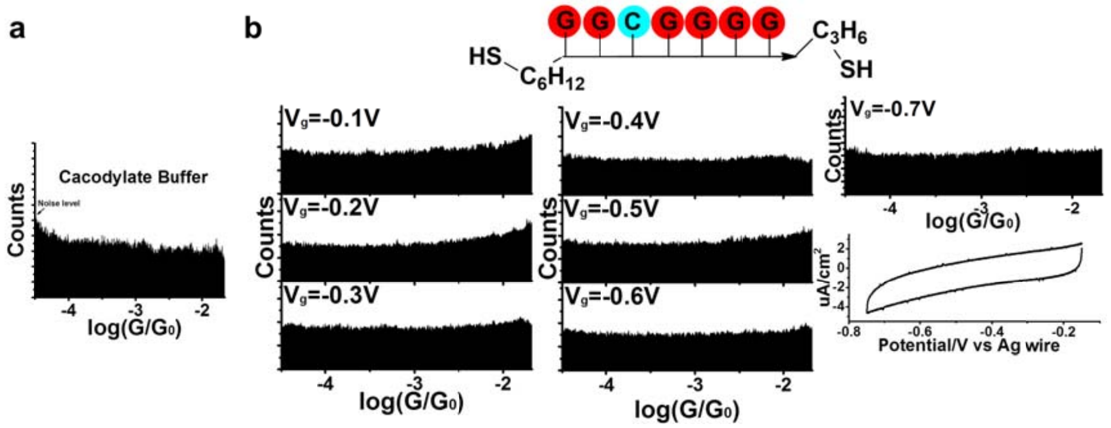

Supplementary Figure 2 | Conductance histograms of cacodylate buffer, and ssDNA with two thiolate linkers at different gate voltages with the cyclic voltammogram (CV) as the control experiments. a, Conductance histogram of cacodylate buffer. No peaks in the conductance histogram detected within the conductance range, ruling out the possibility of solvent-based molecular junction formation. The noise level is due to the lower limit of the current amplified in the STM scanner. **b,** Structure and conductance histograms under different gate voltages with the CV for ssDNA with two thiolate linkers. Note that the CV does not show redox peaks. Conductance histograms also do not have any peaks either because the conductance of ssDNA is below the measurable range.

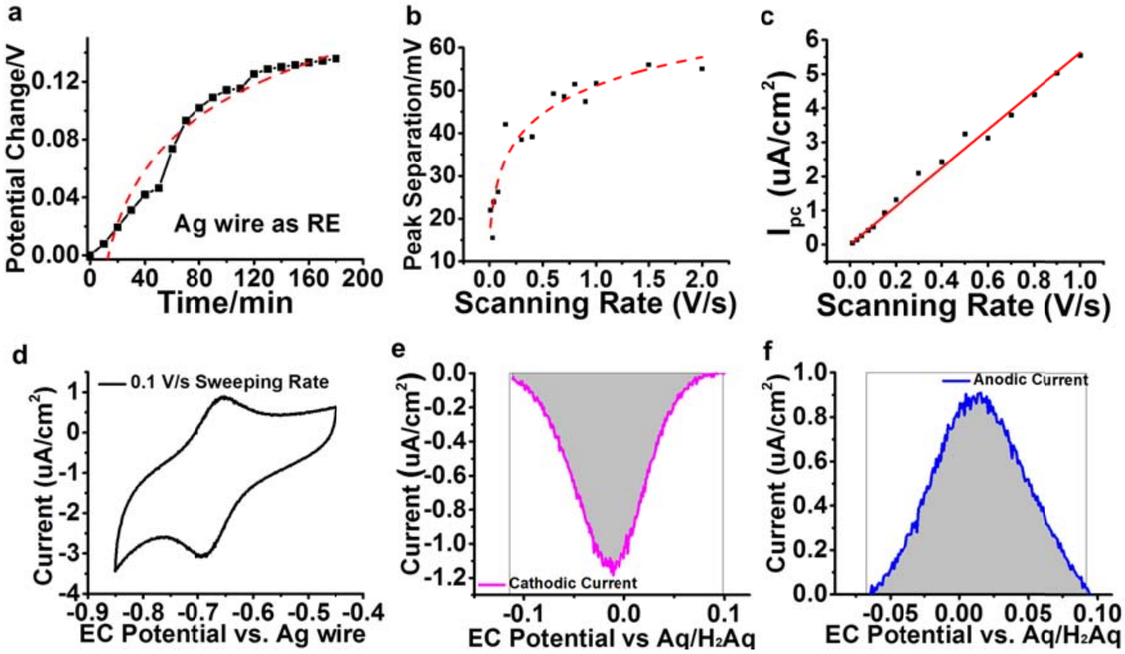

Supplementary Figure 3 | Cyclic voltammogram study of the Aq-DNA modified Au substrate. **a,** Plot of the redox potential changes vs. time, where the red line is a guide to eyes. In the electrochemical gate controlled conductance measurement, the use of silver quasi reference electrode introduced variations in the actual applied gate voltages. To study the variations, we recorded the cyclic voltammograms for Aq-DNA modified Au surface for 3 hours (all the conductance measurements on the Au surface can be finished within 3 hours) in 10 minutes intervals. We found the redox potential of Aq-DNA shifted to the positive direction with time, from which we concluded that the reference potential shifted to the negative direction. This phenomenon is robust as we can see from the cyclic voltammograms before and after the EC-STM break junction conductance measurements (Supplementary Fig. 4-5). Hence, we used the differences in the redox potential as the errors in the gate voltages (V_g). **b,** Separation between the oxidation and reduction peaks vs. potential sweeping rate. Red line is a guide to eyes. **c,** The cathodic peak current I_{pc} versus potential sweeping rate. The linear relation confirms that the redox peaks were due

surface bound Aq-DNA molecules. Fitting the data with a linear dependence (red line) provides a surface coverage of 1.48±0.03 pmol·cm^{-2}. Ag/AgCl reference electrode was used in **b** and **c**. **d,** Cyclic voltammogram obtained with 0.1 V·s^{-1} sweeping rate, from which a cathodic peak and anodic peak were determined after baseline correction. Note: A silver wire was used as quasi-reference electrode to be consistent with the STM break junction experiments. The redox potential was determined by averaging the cathodic peak and anodic peak. **e,** Cathodic peak in the CV. **f,** Anodic peak in the CV. Note: The potential in **e** and **f** are quoted with respect to the redox potential of anthraquinone. The areas of the cathodic and anodic peaks provide the number of molecules participated in the redox reaction.

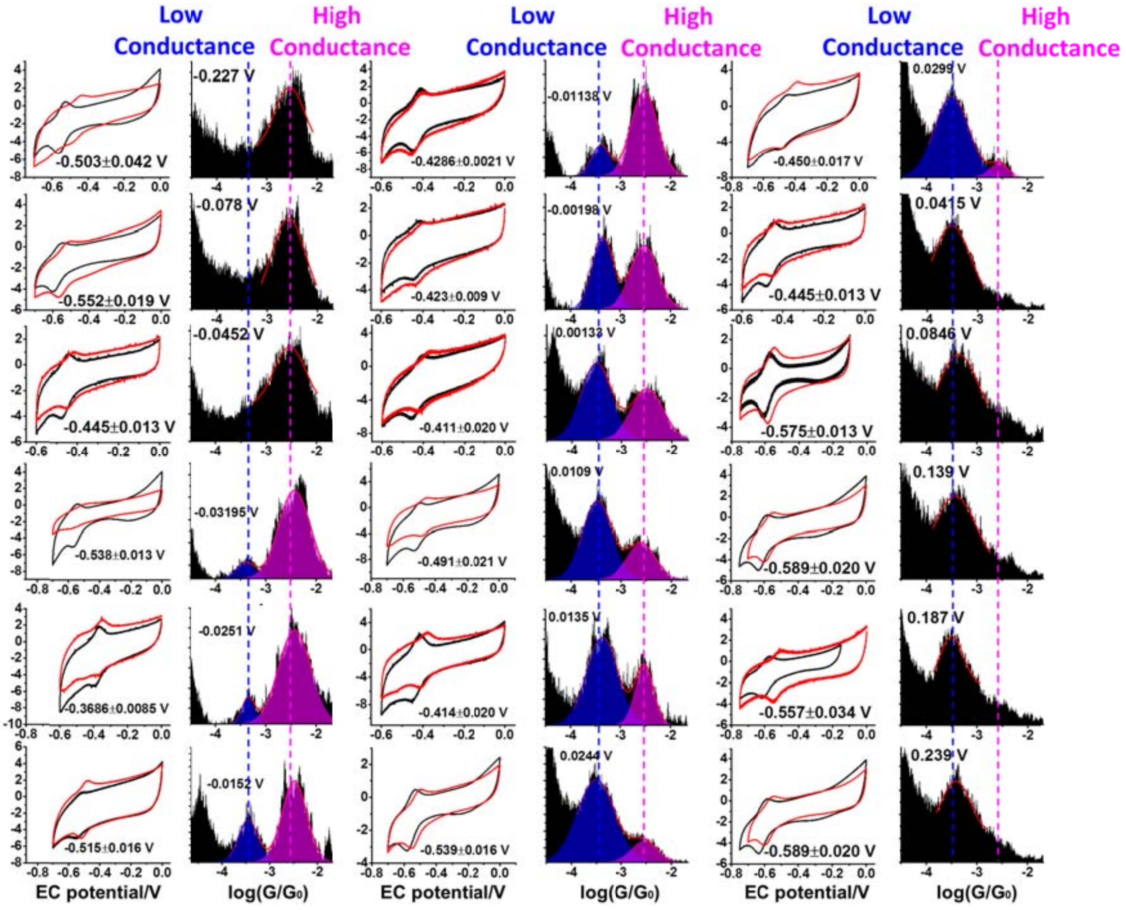

Supplementary Figure 4 | Cyclic voltammograms (CVs) before and after electrochemical gate controlled conductance measurements and the corresponding conductance histograms of Aq-DNA. Note that the applied gate voltage was shown in each conductance histogram. The black and red curves are the CVs before and after the conductance measurement, respectively. Redox potential of Aq/H_2Aq with respect to the Ag wire quasi-reference electrode is shown in the CV. The shifts in the redox peaks were taken as error bars in the gate voltages. When the applied gate voltage is far away from the redox potential of Aq, only one peak was given in each of the conductance histograms. When the applied gate voltage is close to the redox potential of Aq, two peaks were given in each of the conductance histograms.

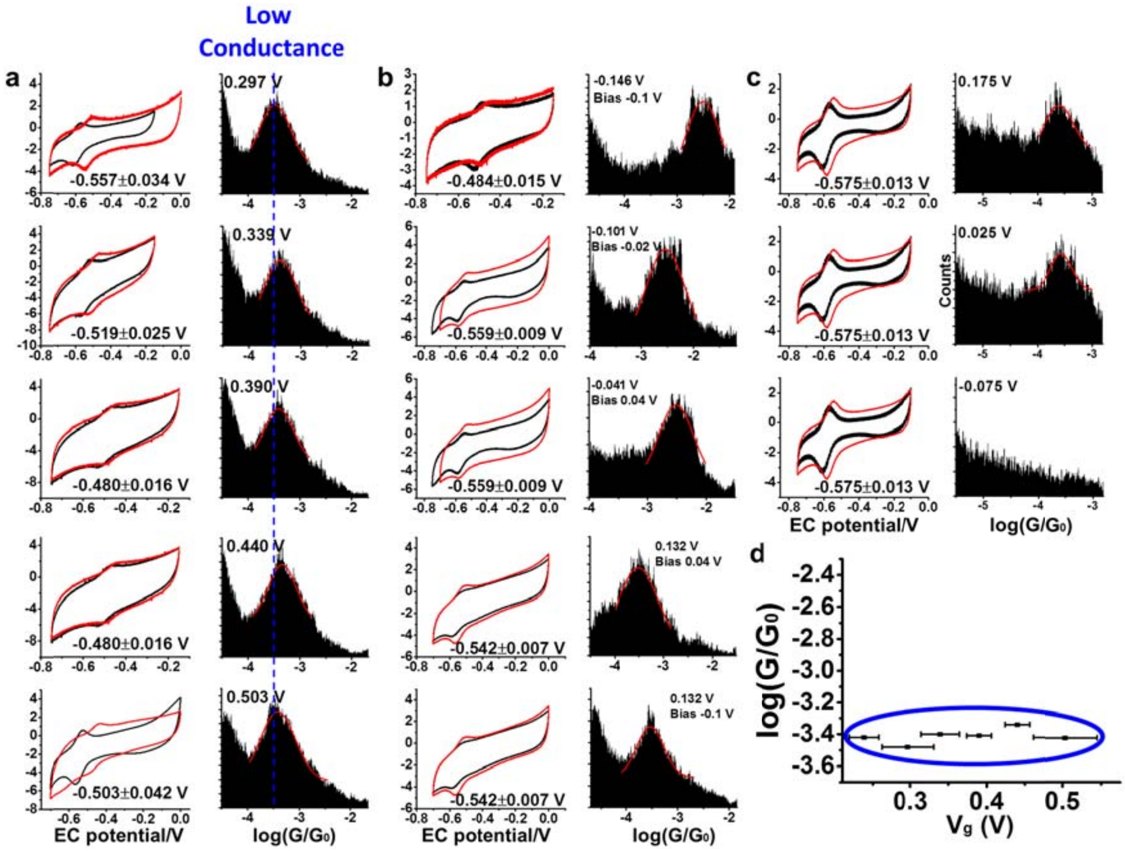

Supplementary Figure 5 | Cyclic voltammograms (CV) of Aq-DNA before and after electrochemical gate controlled conductance measurements, the corresponding conductance histograms and the peak position vs. gate voltage plot for Aq-DNA. **a,** The applied gate voltage was shown in each conductance histogram. The black and red curves are the CVs before and after the conductance measurement, respectively. Redox potential of Aq/H_2Aq with respect to the Ag wire quasi-reference electrode is shown in the CV. The shifts in the redox peaks were taken as error bars in the gate voltages. **b,** The bias voltages (not 5 mV) are shown in the conductance histograms. These show that the conductance of low and high conductance peak is not dependent on bias voltage or bias polarity. **c,** The applied gate voltages were marked in the conductance histograms. There are no conductance peaks in the range from ~$10^{-5.5}$ to ~10^{-4} G_0. Note that the whole

conductance range is from $\sim 10^{-5.5}$ to $\sim 10^{-2.8}$ G_0. **d,** The low onductance of Aq-DNA does not change when gate voltage is from 0.2 to 0.55 V.

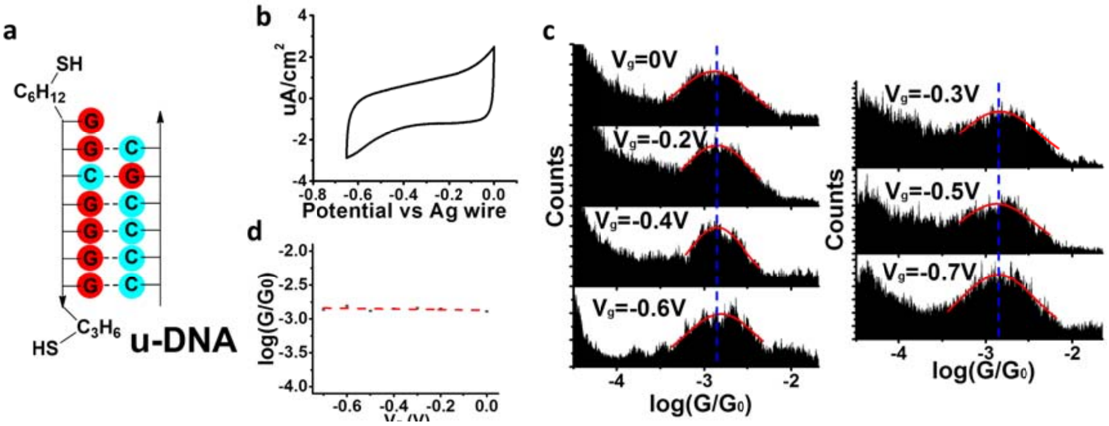

Supplementary Figure 6 | Conductance histogram at different applied gate voltages, cyclic voltammograms (CV) and conductance vs. gate voltage plot for u-DNA as the control experiments. a, Structure of u-DNA. **b,** CV does not show redox peaks. **c,** Conductance histogram under different gate voltages where the *V*g is with respect to the Ag wire quasi-reference electrode. **d,** Conductance does not change with respect to the gate voltage. The red line is a guide to eyes.

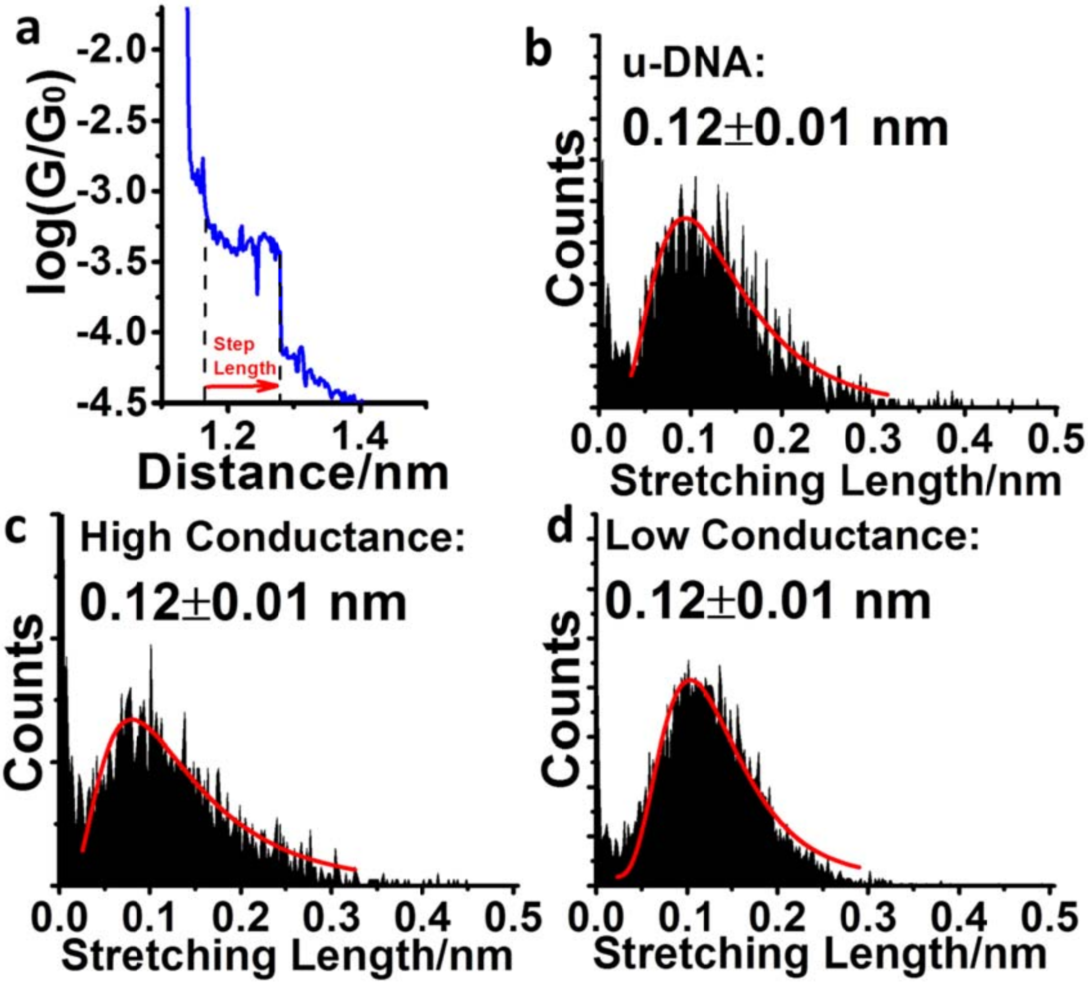

Supplementary Figure 7 | Step length analysis of Aq-DNA and u-DNA. a, Step length is the distance over which a molecular junction can be stretched before breakdown, which corresponds to the length of the conductance plateau in the current-distance traces. This length is represented by the distance between the two black dash lines. The absolute value of the distance on the x-scale means the movement of the piezo scanner in the STM, which does not have any physical meaning to the molecular junctions. **b-d,** Step length histograms for u-DNA, high conductance state of Aq-DNA and low conductance state of

Aq-DNA. The peaks were fitted with a lognormal distribution and the mean values were taken as the step length. Errors were the fitting errors.

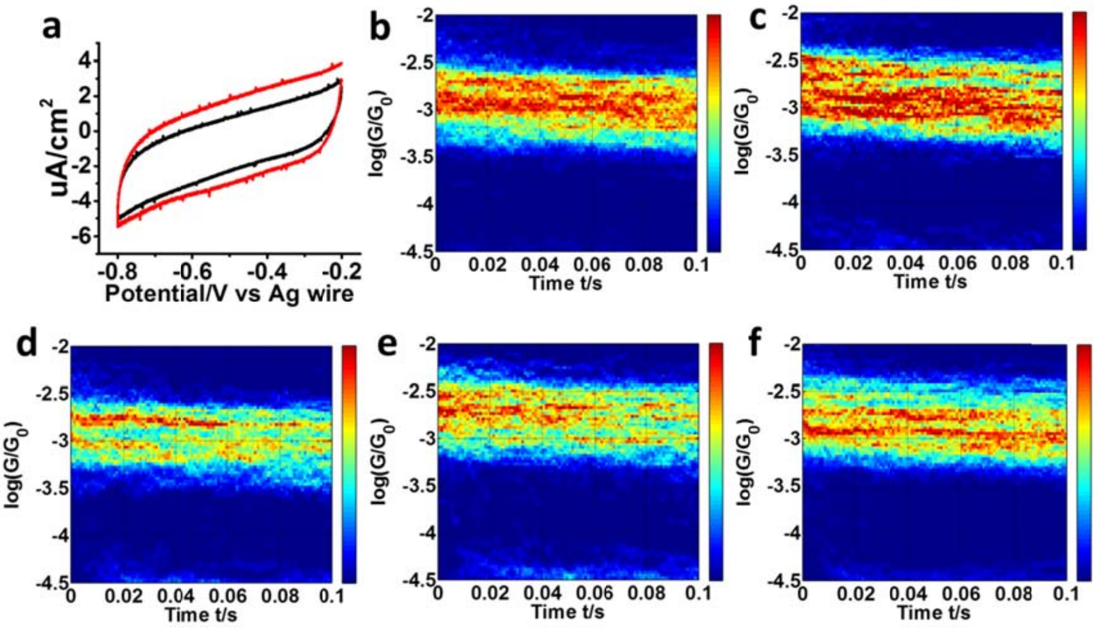

Supplementary Figure 8 | 2D conductance-time (*G-t*) histogram at different gate voltages and cyclic voltammograms (before and after the conductance measurements) for u-DNA as the control experiments. a, Cyclic voltammograms (before and after the conductance measurements) for u-DNA, showing no redox peaks. **b-f,** 2D *G-t* histograms at V_g of -0.65 V, -0.60 V, -0.55 V, -0.50 V and -0.45 V, showing only one band, which is consistent with the conductance measurement at difference gate voltages in Supplementary Fig. 6.

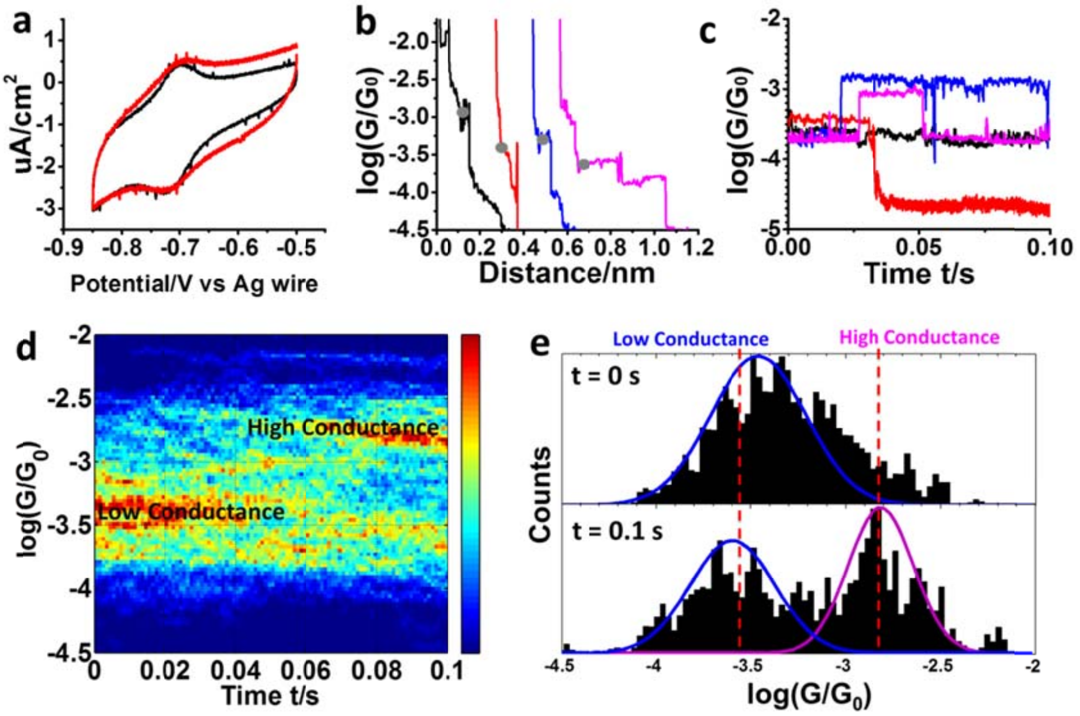

Supplementary Figure 9 | 2D *G*-*t* histogram study started with the low conductance state of Aq-DNA. a, cyclic voltammograms (before and after the conductance measurements). **b,** Conductance-distance trace with plateau at low conductance was detected. Then the tip was fixed in position (grey points) and held for 0.1 s while the current was recorded. **c,** Similar to Figure 5b, three major types of conductance-time (*G*-*t*) traces were observed (black, red and blue traces). A few of the traces have multiple switching events occurring (magenta trace). **d,** 2D *G*-*t* histogram showing two bands. As the low conductance band fades away with time, and the high conductance starts to appear. **e,** 1D conductance histograms at t = 0 and t = 0.1 s, showing the switching from low conductance state to high conductance state.

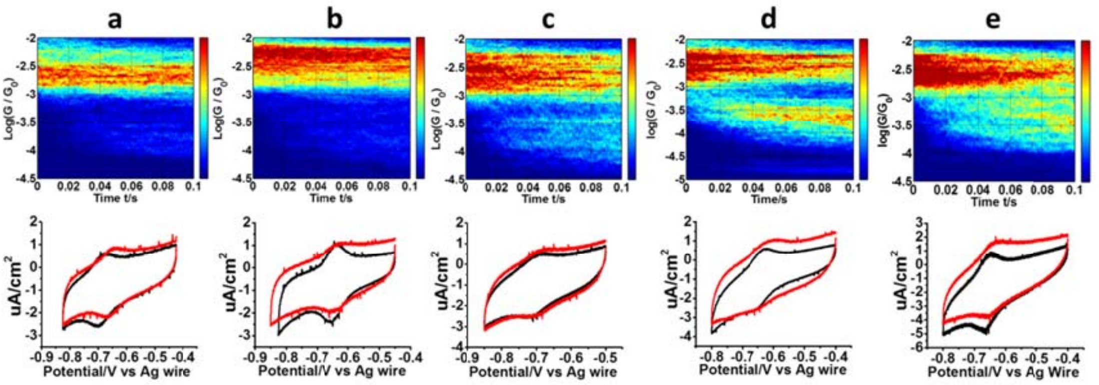

Supplementary Figure 10 | 2D conductance-time (*G-t*) histogram at different gate voltages and cyclic voltammograms (before and after the conductance measurements) for Aq-DNA. a, V_g = -30±17 mV**. b,** V_g = -21±7 mV**. c,** V_g = -13±8 mV. **d,** V_g = -0±5 mV. **e,** V_g = 9±10 mV. Gate voltage is vs. the redox potential of Aq/H_2Aq in the CV. Notice that there will be more switching events when gate voltage is more positive.

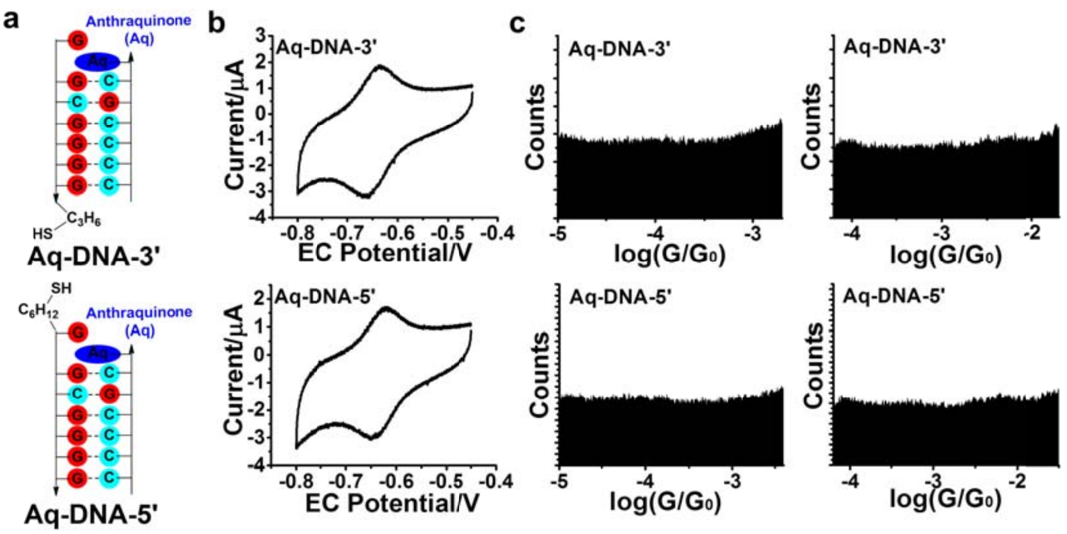

Supplementary Figure 11 | Structure, CV and conductance histograms of monothiol Aq-DNA. a, Structure of Aq-DNA with monothiol at 3'-end or 5'-end, named Aq-DNA-3' or Aq-DNA-5' respectively. **b,** CV for Aq-DNA-3' and Aq-DNA-5', showing the redox peak of Aq moiety. **c,** Conductance histograms of Aq-DNA-3' and Aq-DNA-5' showing no peaks, which indicates the need of both thiol groups to bridge Aq-DNA between the two electrodes. Note: The only possible binding geometry is the upright geometry as shown in Fig. 1a.

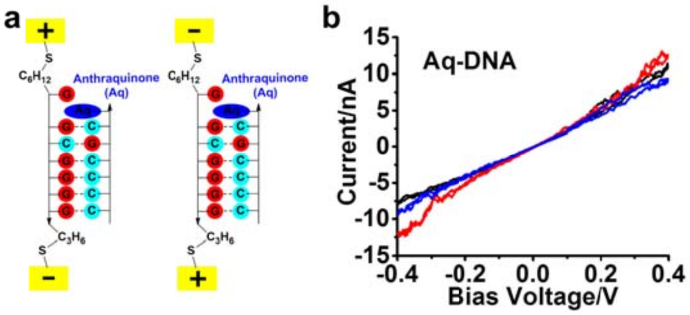

Supplementary Figure 12 | Current-voltage (*I-V*) characteristics of Aq-DNA. a, Two different voltage polarities. **b,** Representative *I-V* curves for Aq-DNA, showing linear behavior. **c,** 2-dimensional conductance-voltage (*G-V*) constructed by thousands of *I-V* curves, showing that the conductance does not depend on bias voltage or its polarity.

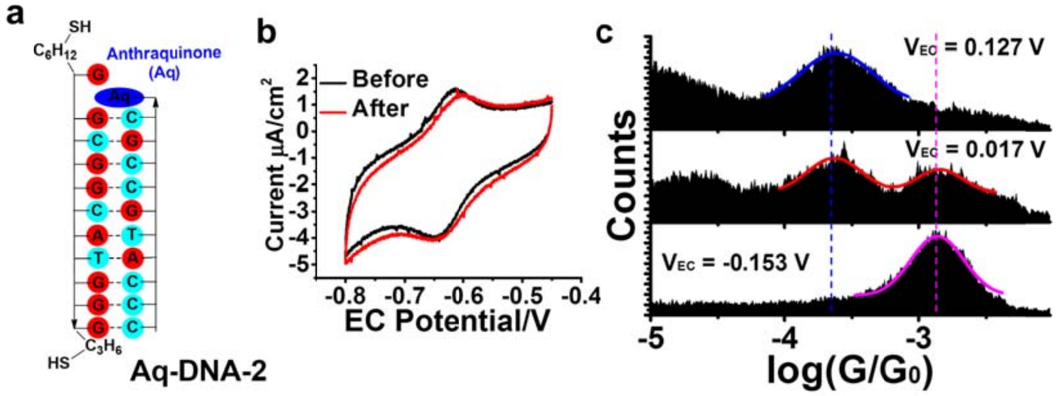

Supplementary Figure 13 | Structure, CV and conductance histogram of Aq-DNA-2 with 10 base pairs. a, Structure of Aq-DNA-2. **b,** CV before and after the conductance measurements, showing the redox peak of Aq moiety. **c,** Conductance histograms under different electrochemical gate voltages. When gate voltage is above or below the redox potential, only low conductance peak or high conductance peak shows up, respectively. When gate voltage is close to the redox potential, both peaks show up. The low and high

conductance values are 2.5×10^{-4} G_0 and 1.4×10^{-3} G_0, respectively, smaller than the low and high conductance values for Aq-DNA. These findings demonstrate that the measured charge transport involves the DNA sequences and one can tune the conductance values of the two states by changing the DNA sequence.

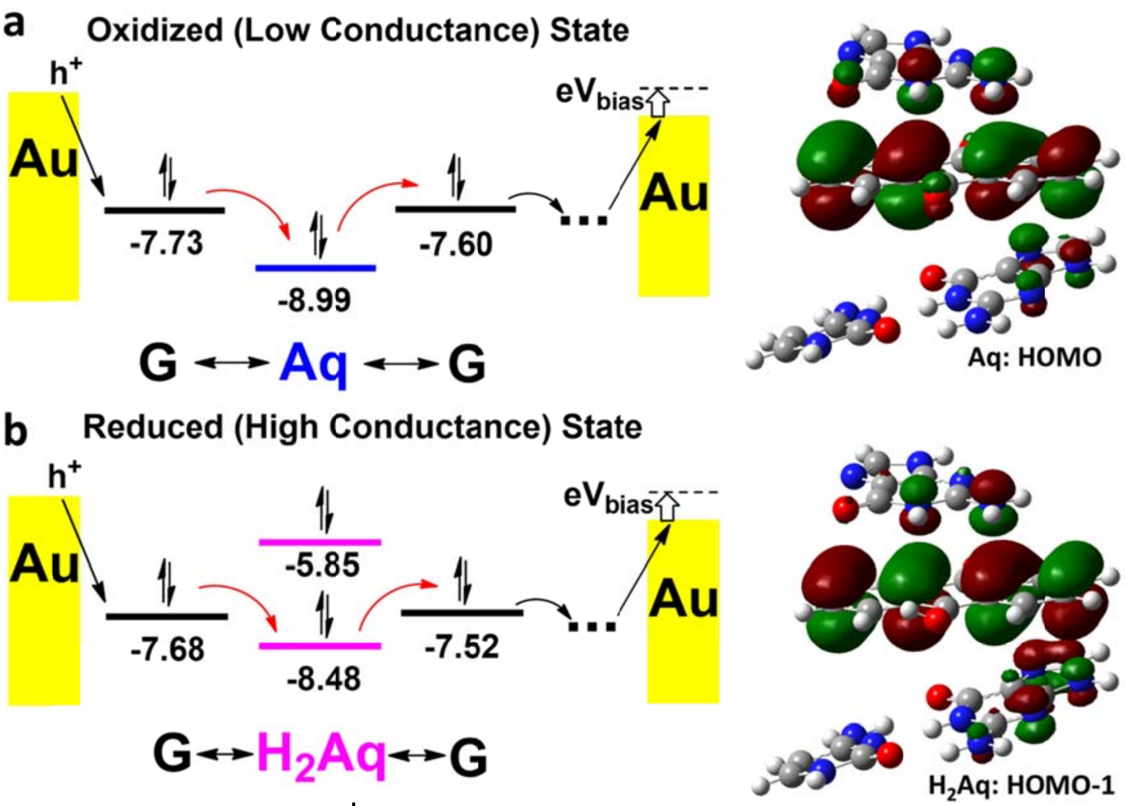

Supplementary Figure 14 | Energy diagram for the charge transport and molecular orbital spatial distribution in a) oxidation state and b) reduction state at ZINDO/S semiempirical level. Similar to Fig. 6 obtained from DFT calculations at the M06-2X/6-311+G(p,d) level of theory, the HOMO-1 of H_2Aq is closer to the HOMO levels of Guanines comparing to the HOMO of Aq. Molecular orbital spatial distribution indicates the orbital main localized on the Aq moiety for both cases.

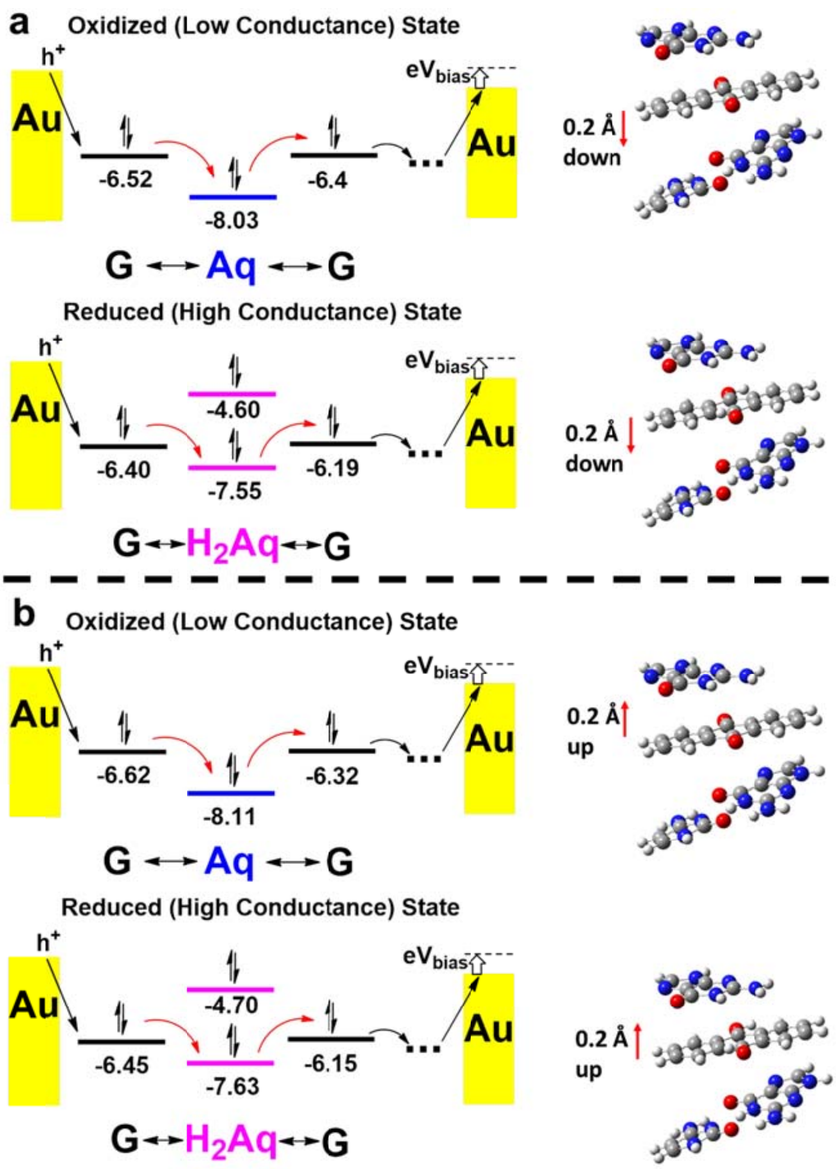

Supplementary Figure 15 | Energy diagram for the charge transport and molecular orbital spatial distribution in both states when modifying the position of Aq moiety. **a,** The Aq and H_2Aq were moved down for 0.2 Å. **b,** The Aq and H_2Aq were moved up for 0.2 Å. Results are from DFT calculations at the M06-2X/6-311+G(p,d) level of theory. Both energy level alignments and spatial distribution are weakly dependent on the position of Aq moiety.

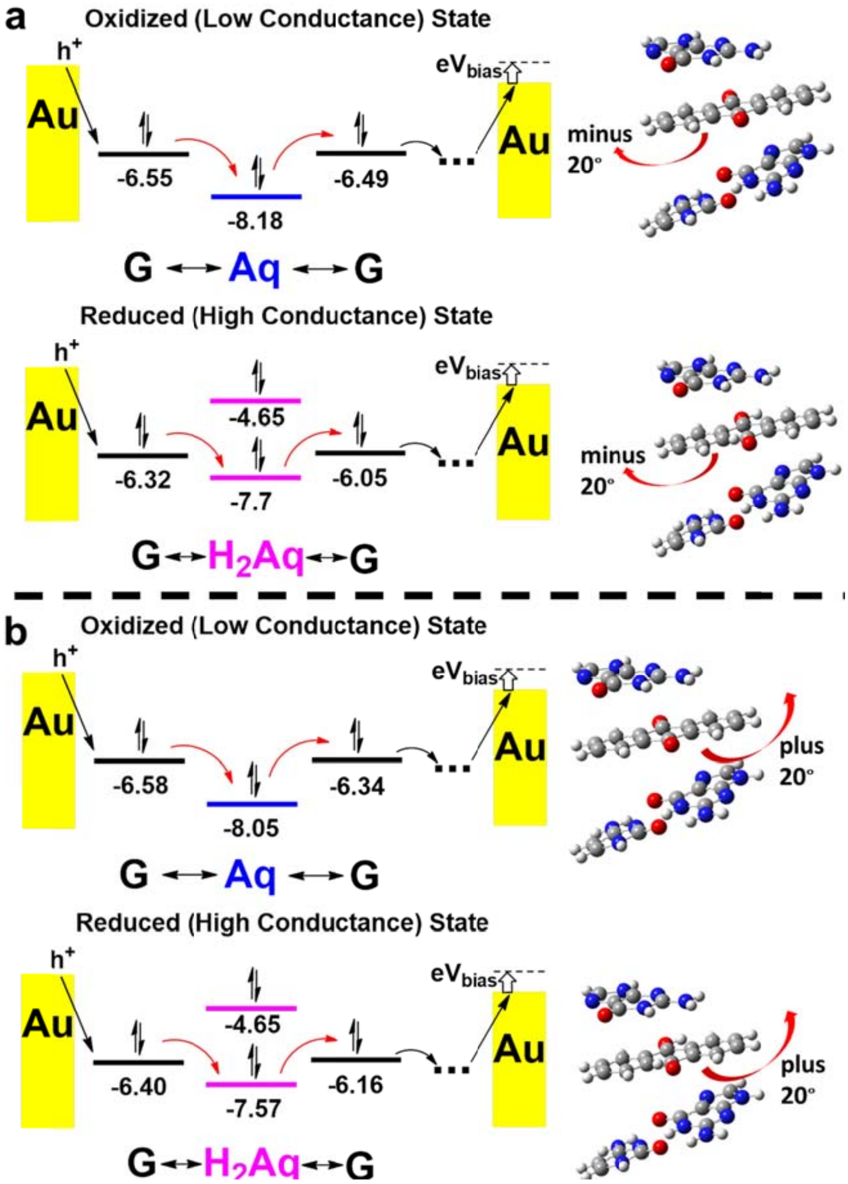

Supplementary Figure 16 | Energy diagram for the charge transport and molecular orbital spatial distribution in both states when rotating the Aq moiety. a, The Aq and H_2Aq were moved clockwise for 20º. **b,** The Aq and H_2Aq were moved counterclockwise for 20º. Results are from DFT calculations at the M06-2X/6-311+G(p,d) level of theory. Both energy level alignments and spatial distribution are weakly dependent on the angle of Aq moiety.

Supplementary Table 1 | Conductance values with experimental errors under different electrochemical gate voltages.

Relative Gate Voltage V_g/V	Error in Gate Voltage/V	Conductance Value/10^{-4} G_0	Error in Conductance Value/10^{-4} G_0
-0.0114	0.0021	30.2	0.7
-0.0114	0.0021	4.0	0.1
0.014	0.020	30.2	0.7
0.0144	0.020	4.4	0.1
0.0013	0.020	33.1	0.8
0.0013	0.020	3.1	0.1
0.024	0.011	29.5	1.4
0.024	0.011	3.1	0.1
-0.045	0.013	29.5	0.7
0.0414	0.0022	3.1	0.1
-0.0020	0.0087	30.2	0.7
-0.0020	0.0087	4.4	0.1
-0.0251	-0.0026	37.2	0.9
-0.0251	-0.0026	4.5	0.1
-0.032	-0.013	37.2	0.9
-0.032	-0.013	4.0	0.2
0.011	-0.021	25.7	0.6
0.011	-0.021	3.3	0.1
0.030	-0.017	27.5	2.5
0.030	-0.017	3.2	0.1
-0.015	-0.016	33.1	0.8
-0.015	-0.016	3.5	0.1
-0.227	0.042	28.8	0.7
0.139	0.019	3.7	0.1
0.239	0.019	3.8	0.1
-0.078	0.019	26.9	0.6
0.1320	0.0066	3.1	0.1
-0.1010	0.0092	28.8	0.7
0.085	0.013	4.4	0.1
-0.146	0.015	31.6	0.7
0.187	0.034	3.2	0.1
-0.0410	0.0092	30.9	0.7
0.503	0.042	3.8	0.1
0.239	0.019	3.8	0.1
0.297	0.034	3.3	0.1
0.339	0.025	4.0	0.1
0.390	0.016	3.9	0.1
0.440	0.016	4.6	0.1

Supplementary Table 2 | k_f and k_b values, fitted gate voltages and the applied gate voltages from 2D *G-t* histograms. By fitting the curves in Fig. 5e (The peak area of the high conductance states versus time plot in the 2D *G-t* histograms) with equation (4), we were able to obtain k_f and k_b. Combing these values with equation (3), the fitted gate voltages can be determined to compare with the applied gate voltages.

k_f	k_b	Fitted V_g/mV	Applied V_g/mV
8.49+0.30	4.0+0.9	9+3	9+10
9.78+0.34	10.0+1.0	0+2	0+5
5.65+0.38	15.3+2.2	-13+3	-13+8
4.17+0.37	12.0+2.7	-13+4	-21+7
2.09+0.58	16+9	-25+11	-30+17

Supplementary Table 3 | Experimental error for Aq-DNA and u-DNA when EC gate voltage is off

	1st time	2nd time	3rd time	Result
Aq-DNA	-3.41	-3.41	-3.39	-3.40±0.01
u-DNA	-2.82	-2.83	-2.87	-2.85±0.02

Supplementary Note 1 | Analysis of the relationship between the conductance peak area and the surface coverage of the redox species

To confirm that there are only two conductance states, we checked the conductance ranging from 3.2×10^{-6} to 2.5×10^{-2} G_0 and did not detect any other conductance peaks (see Supplementary Fig. 5l-5n). Also, it is unlikely that more than one DNA molecule could bridge between the tip and substrate, due to repulsion between the negatively charged DNA. Thus we attribute each of the individual current-distance traces in the conductance

histograms to one single DNA molecular junction. In this way, the peak area S in a conductance histogram can be expressed by[2],

$$S = n_j \cdot \frac{Lf}{vU} \quad (1)$$

where n_j is the number of molecular junctions (traces that have plateau), L is the length of the plateau regime, or step length, f is the sampling frequency, v is the pulling rate in $nm \cdot s^{-1}$ and U is the bin number in the conductance histogram. The step lengths L for the two conductance states are the same (Supplementary Fig. 7, consistent with Bruot et al.'s results[3]), so the peak area S is proportional to the number of molecular junctions, n_j.

In STM break junction experiment, n_j can be expressed as:

$$n_j = N \cdot Y \quad (2)$$

Where N is the total current-distance trace collected and Y is the chance of forming a molecular bridge during one current-distance trace. The oxidized state and reduced state were measured under the same circumstance (e.g. same solution, temperature, Au tip and substrate), and their structures only differ by the Aq moiety. Therefore, we believe that the Y is proportional to the surface coverage of the species Γ. Thus ideally the peak area S is proportional to Γ.

Supplementary Note 2 | Switching probabilities of *G-t* trace under different gate voltages

The conductance in the *G-t* trace can only be either in the high conductance state (Aq is in oxidized state) or in the low conductance state (Aq is in reduced state), which can be described as:

$$G = (1-p)\cdot G_{ox} + p\cdot G_{red}\,,\ p = 0 \text{ or } 1. \quad (3)$$

However, the probability of finding a single molecule in the reduced state $P_{\mathrm{eq}}(\mathrm{red})$ or in the oxidized state $P_{\mathrm{eq}}(\mathrm{ox})$ under an equilibrium depends on the gate voltage V_g according to Nernst equation.

$$P_{\mathrm{eq}}(\mathrm{red}) = \frac{1}{1+e^{\frac{nF}{RT}V_g}} \quad (4)$$

$$P_{\mathrm{eq}}(\mathrm{ox}) = \frac{e^{\frac{nF}{RT}V_g}}{1+e^{\frac{nF}{RT}V_g}} \quad (5)$$

Notice that $P_{\mathrm{eq}}(\mathrm{red}) + P_{\mathrm{eq}}(\mathrm{ox}) = 1$. The Equation (4) in the main text describes how the percentage of reduced state changes with respect to time when starting from $P_t(\mathrm{red}) = 1$. As time t goes to infinity, the Equation (4) will eventually become Supplementary Equation (4). This is reasonable as one would expect an equilibrium state being reached after infinite amount of time.

$$\lim_{t\to\infty} P_t(\mathrm{red}) = \lim_{t\to\infty} \frac{k_f e^{-(k_b+k_f)t} + k_b}{k_b + k_f} = \frac{k_b}{k_b + k_f} = \frac{\Gamma_{\mathrm{red}}}{\Gamma_{\mathrm{red}} + \Gamma_{ox}} = P_{\mathrm{eq}}(\mathrm{red}) \quad (6)$$

Supplementary Note 3 | Experimental errors of conductance measurements

To examine the reproducibility of the conductance measurements in the conductance measurements when electrochemical gate voltage was off, the experiments for u-DNA and Aq-DNA were repeated three times, each consisting of ~4000 curves. The results were listed in the table below, showing the reproducibility of the conductance measurements. The experimental error was calculated by: $\sigma = \sqrt{\frac{1}{N}\sum_{i=1}^{N}\left(x_i - \mu\right)^2}$, where N is the number of sets, x_i is the peak position in each individual set of experiment (on a logarithm scale) and μ is the peak position obtained by compiling all the 3 histograms (Supplementary Table 3). For conductance measurements under different gate voltages, we used the fitting errors (Gaussian fitting) in each of the histograms at different gate voltages as the experimental errors[4], as shown in Fig. 3g. The broad distribution (the width in the Gaussian fit) in the conductance histogram is an inherent property of single molecule measurement originated from the variation in the molecule-electrode contact coupling, and dependence of the conductance on the couplings[5, 6], rather than an experimental error.

Supplementary References:

1. Patra, A. & Richert, C. High Fidelity Base Pairing at the 3'-Terminus. *J. Am. Chem. Soc.* **131**, 12671-12681 (2009).
2. Arielly, R., Vadai, M., Kardash, D., Noy, G. & Selzer, Y. Real-Time Detection of Redox Events in Molecular Junctions. *J. Am. Chem. Soc.* **136**, 2674-2680 (2014).
3. Bruot, C., Xiang, L., Palma, J.L. & Tao, N. Effect of Mechanical Stretching on DNA Conductance. *ACS Nano* **9**, 88-94 (2015).
4. Richter, P. Estimating errors in least-squares fitting. *Telecommun. Dat. Acqu. Progres. Rep.* **42-122**, 107-137 (1995).
5. Quan, R., Pitler, C.S., Ratner, M.A. & Reuter, M.G. Quantitative Interpretations of Break Junction Conductance Histograms in Molecular Electron Transport. *ACS Nano* **9**, 7704-7713 (2015).

6. Malen, J.A. *et al.* The Nature of Transport Variations in Molecular Heterojunction Electronics. *Nano Lett.* **9**, 3406-3412 (2009).
